# Supplementary material for: How information about perpetrators’ nature and nurture influences assessments of their character, mental states, and deserved punishment
Source: PLoS One. 2019 Oct 22;14(10):e0224093. doi: 10.1371/journal.pone.0224093 (PMC6804977; doi:10.1371/journal.pone.0224093)
Supplement: S1 File — (DOCX) [file pone.0224093.s001.docx]

**How Information about Perpetrators’ Nature and Nurture Influences Assessments of their Character, Mental States, and Deserved Punishment**

**Supplementary Materials**

**Study 1**

***Essentialism Measures***

Study 1 employed an Essentialism Scale that was adapted from a combination of two previously validated scales. Items 1–4 were taken from Keller (2005), while Items 5–8 were taken from Rangel & Keller (2011).

1. I think the main reason why parents and children are so alike in behavior and character is that they possess a shared genetic inheritance.
2. I believe that children inherit many of their personal traits from their parents.
3. I am convinced that very few behavioral traits of humans can be traced back to their genes. (R)
4. I think that genetic predispositions have little influence on a person’s personality characteristics. (R)
5. An individual’s personality often reveals the social origin of the person. (R)
6. I am convinced that the social background a person comes from is strongly reflected in the person’s character. (R)
7. It is possible to make fairly good predictions concerning the personality traits of an individual based on the knowledge of his or her social origin. (R)
8. I think the character of individuals is only to a very limited degree determined by their social origin.

**Study 2**

***Essentialism Measure***

This study used the Belief in Genetic Determinism (BGD) Scale (Keller, 2005) to measure essentialism. This measure includes the following items (responses were recorded on a 7-point scale ranging from 1: not at all true to 7: completely true).

1. I think the chief reason why parents and children are so alike in behavior and character is that they possess a shared genetic inheritance.
2. In my opinion, alcoholism is caused primarily by genetic factors.
3. I think that differences between men and women in behavior and personality are largely determined by genetic predisposition.
4. I believe that children inherit many of their personal traits from their parents.
5. In my view, the development of homosexuality in a person can be attributed to genetic causes.
6. I am convinced that very few behavioral traits of humans can be traced back to their genes. (R)
7. I believe that many talents that individuals possess can be attributed to genetic causes.
8. I think that the upbringing by parents and the social environment have far greater significance for the development of abilities and personal traits than genetic predispositions. (R)
9. I believe that many differences between humans of different skin color can be attributed to differences in genetic predispositions.
10. I think that genetic predispositions have little influence on a person’s personality characteristics. (R)
11. In my view, many forms of human behavior are biologically determined and can therefore be seen as instinctual.
12. The fate of each person lies in his or her genes.
13. I am of the opinion that intelligence is a trait that is strongly determined by genetic predispositions.
14. I believe that genetic predispositions have no influence whatsoever on the development of intellectual abilities. (R)
15. I am convinced that the analysis of the genetic predispositions of an embryo allows good predictions as to which characteristic and abilities the child will develop.
16. I think the genetic differences between Asians and Europeans are an important cause for the differences in abilities between individuals from these groups.
17. I think that twins, because of the identical genetic predispositions, will be very similar in their behavior even if they were adopted and raised in different families.
18. I believe that an analysis of my genetic predispositions will allow a trained scientist to predict many of my abilities and traits without having any personal knowledge of me.

***Additional Analyses***

There was no interaction between Genes and Environment for any of the variables: Intention, *F*(1, 132) = 1.945, *p* = .165, *η^2^_p_* = .015; Free Will, *F*(1, 132) = 2.611, *p* = .108, *η^2^_p_* = .019; Character, *F*(1, 132) = .076, *p* = .784, *η^2^_p_* = .001; Punishment, *F*(1, 132) = .314, *p* = .576, *η^2^_p_* = .002. There was also no interaction between Genes and the type of Transgression (Harm/Purity) for any of the variables: Intention, *F*(1, 132) = 2.761, *p* = .099, *η^2^_p_* = .020; Free Will, *F*(1, 132) = 1.738, *p* = .190, *η^2^_p_* = .013; Character, *F*(1, 132) = .898, *p* = .345, *η^2^_p_* = .007; Punishment, *F*(1, 132) = .149, *p* = .700, *η^2^_p_* = .001. Additionally, there was also no interaction between Environment and type of Transgression (Harm/Purity) for any of the variables: Intention, *F*(1, 132) = 2.860, *p* = .093, __ = .021; Free Will, *F*(1, 132) = .043, *p* = .837, *η^2^_p_* = .000; Character, *F*(1, 132) = .236, *p* = .628, *η^2^_p_* = .002; Punishment, *F*(1, 132) = .079, *p* = .779, *η^2^_p_* = .001. Finally, there was no three-way interaction between Genes, Environment, and type of Transgression for any of the variables: Intention, *F*(1, 132) = .745, *p* = .390, *η^2^_p_* = .006; Free Will, *F*(1, 132) = 1.429, *p* = .234, *η^2^_p_* = .011; Character, *F*(1, 132) = .011, *p* = .918, *η^2^_p_* = .000; Punishment, *F*(1, 132) = .478, *p* = .490, *η^2^_p_* = .004.

**Study 3**

***Essentialism Measure***

In addition to the BGD Scale from Study 2, this study additionally used a scale by Bastian & Haslam (2008) to assess essentialism. This scale includes the following items (on which participants indicated how strongly they agreed or disagreed with the statements “about aspects of people generally” on a scale of 1: strongly disagree to 6: strongly agree).

1. The boundaries that define the differences between people are clear-cut.
2. A person either has a certain attribute or they do not.
3. Everyone is either a certain type of person or they are not.
4. There are different ‘types’ of people and those ‘types’ can be easily defined and are relatively clear-cut.
5. The kind of person someone is, is clearly defined, they either are a certain kind of person or they are not.
6. People fall into distinct personality ‘types’.
7. Generally speaking, once you know someone in one or two contexts it is possible to predict how they will behave in most other contexts.
8. It is possible to know about many aspects of a person once you become familiar with a few of their basic traits.
9. When getting to know a person it is possible to get a picture of the kind of person they are very quickly.
10. Knowing about a few of the basic traits a person has can lead to accurate predictions of their future behaviour.
11. Although a person may have some basic identifiable traits, it is never easy to make accurate judgments about how they will behave in different situations. (Reversed)
12. There are different types of people and with enough scientific knowledge these different ‘types’ can be traced back to genetic causes.
13. Whether someone is one kind of person or another is determined by their biological make-up.
14. With enough scientific knowledge, the basic qualities that a person has could be traced back to, and explained by, their biological make-up.
15. The kind of person someone is can be largely attributed to their genetic inheritance.

***Additional Analyses***

There was no interaction between Genes and Environment for any of the variables: Intention, *F*(1, 140) = .499, *p* = .481, *η^2^_p_* = .004; Free Will, *F*(1, 140) = .080, *p* = .778, *η^2^_p_* = .001; Character, *F*(1, 140) = .570, *p* = .452, *η^2^_p_* = .004; Punishment, *F*(1, 140) = 1.241, *p* = .267, *η^2^_p_* = .009. There also was no interaction between Genes and the type of Transgression (Accidental Harm/ Luck) for any of the variables: Intention, *F*(1, 140) = 2.194, *p* = .141, *η^2^_p_* = .015; Free Will, *F*(1, 140) = 2.041, *p* = .155, *η^2^_p_* = .014; Character, *F*(1, 140) = .006, *p* = .940, *η^2^_p_* = .000; Punishment, *F*(1, 140) = .513, *p* = .475, *η^2^_p_* = .004. Additionally, there was no interaction between Environment and type of Transgression (Accidental Harm/Luck) for any of the variables: Intention, *F*(1, 140) = 2.972, *p* = .087, *η^2^_p_* = .021; Free Will, *F*(1, 140) = .409, *p* = .524, *η^2^_p_* = .003; Character, *F*(1, 140) = 2.803, *p* = .096, *η^2^_p_* = .020; Punishment, *F*(1, 140) = .022, *p* = .882, *η^2^_p_* = .000. Finally, there was no three-way interaction between Genes, Environment, and type of Transgression for any of the variables: Intention, *F*(1, 140) = 1.247, *p* = .266, *η^2^_p_* = .009; Free Will, *F*(1, 140) = .508, *p* = .477, *η^2^_p_* = .004; Character, *F*(1, 140) = .448, *p* = .504, *η^2^_p_* = .003; Punishment, *F*(1, 140) = .011, *p* = .918, *η^2^_p_* = .000.
