# Supplementary material for: How information about perpetrators’ nature and nurture influences assessments of their character, mental states, and deserved punishment
Source: PLoS One. 2019 Oct 22;14(10):e0224093. doi: 10.1371/journal.pone.0224093 (PMC6804977; doi:10.1371/journal.pone.0224093)
Supplement: Attachment — Submitted filename: Response to Reviewers R1.docx [file pone.0224093.s002.docx]

Franklin & Marshall College

Department of Psychology

P.O. Box 3003

Lancaster, PA 17604-3003

Professor Valerio Capraro

Academic Editor, *PLOS One*

September 12, 2019

Dear Dr. Capraro,

We have attached a revision of our manuscript, “How Information about Perpetrators’ Nature and Nurture Influences Assessments of their Character, Mental States, and Deserved Punishment” (PONE-D-19-15890). We were thrilled to receive such positive and helpful feedback from you and the four reviewers, and we thank you and the reviewers for such detailed and insightful advice. We have edited our manuscript in accordance with these comments, and we have submitted both a clean copy and a copy where we have tracked all of our revisions.

As you suggested, we gave particular care to the comments from Reviewer 2, especially the suggestion that our three studies are underpowered. Although the reviewer suggests that we re-run the studies with a larger sample, we have instead presented power analyses indicating that we had sufficient power to detect true medium-sized effects in all three studies (Study 1: power = .825; Study 2: power = .836; Study 3: power = .856). Therefore, even in light of the current replicability crisis, there is no indication that our samples were problematically small – particularly given that we were interested in whether information about genes and environments had meaningful, medium-to-large, effects on punishment.

We have also carefully addressed all other comments. Below, please find a detailed description of the changes that we have made in response to each of the reviewers’ comments. The reviewers’ suggestions are **bolded**, and our responses are indented and *italicized*.

Sincerely,

Julianna M. Lynch, Franklin & Marshall College

Jonathan D. Lane, Vanderbilt University

Colleen M. Berryessa, Rutgers University

Joshua Rottman, Franklin & Marshall College ([jrottman@fandm.edu](mailto:jrottman@fandm.edu))

--------------------------

**Reviewer #1:**

**I suggest making some minor changes to the method sections. In Study 1 it would be helpful to first introduce the dependent measures in the context of explaining the proposed composite measures. A brief overview of the phases of the procedure should be provided in the text along with the flowchart so that readers will be able to understand what “the same transition” refers to without having to look at the chart. Also, the way the participants section is written in Study 1 implies that “adult participants” will be followed by a description of child participants.**

*We agree with the Reviewer that making these adjustments to the Methods will improve overall clarity about the design and procedure of the study. When introducing the dependent measures, we now specify that Question 1 formed the Intention measure, Question 2 formed the Free Will measure (p. 7: lines 181–185), Questions 3–5 formed the Punishment composite measure (pp. 7–8: lines 185–190), and Questions 6–7 formed the Character composite measure (p. 8: lines 190–194). To clarify the phases of the procedure, we have provided a brief summary (pp. 6–7: lines 164–173) about each phase of the vignette: First a description of birth parents was provided, then participants read the switched-at-birth transition, followed by the background of the biologically unrelated parents, and lastly the description of the moral transgression was provided. Finally, to avoid confusion, we deleted “adult” from the description of the participants (this is indeed unnecessary, given that we include information about the age of our sample).*

**Punishment is defined as attempting to alter or reform the bad behavior, either socially or legally. However, it can also send messages that have nothing to do with the person who committed the transgression, either as a signal to others who might be thinking of committing a crime, or as a signal to victims that their suffering matters (which can help to explain why outcomes matter so much).**

*This is an important point. We have now noted in the paper that a primary reason for Genes and Environments having null effects on punishment is that a focus on extenuating circumstances would degrade the signaling and teaching functions of punishment: “One explanation for the null effect of background factors on punishment assessments is that punishment decisions (particularly as compared to assessments of moral wrongness) rely heavily on the outcomes of actors’ behaviors [7]. Empirical and theoretical work has indicated that the signaling and pedagogical functions of punishment are most effective when punishment decisions are primarily driven by the presence of direct consequences, rather than by invisible factors such as a person’s internal dispositions [8]” (p. 25: lines 581–586).*

**In the description of Study 2, it is unclear whether people would accept that genetic influences relating to being nice or mean could have implications for running around on the playground naked and shouting dirty words.**

*We are unsure why people would be more skeptical of genetic influences contributing to deviant behaviors in the form of impurity as compared to genetic influences contributing to deviant behaviors in the form of harm. Crucially, however, participants need not think that the protagonists’ genetic backgrounds “make them” perform highly specific actions in order for these backgrounds to have an exculpatory (or aggravating) effect on punishment decisions.*

**Also, in Study 2, I wondered whether the difference in punishment recommendations between the harm and purity transgression types is really about harm, or about how severe the transgressions are perceived to be. What if you replaced the harm transgression with one in which the level of harm was likely to be small, such as hurting someone’s feelings by calling him stupid, or spilling someone’s juice? It might also matter if the harm were incidental to some other activity.**

*While we chose a harmful behavior that was much less severe than behaviors that are typically studied to discern the relevance of background circumstances for punishment (e.g., murder), it is true that the purity transgressions were perceived to be less severe. (This is often the case for purity violations; see Gray & Keeney, 2015 and Graham, 2015.) We do not see any reason to expect that we would have found different results with less severe harms, but we now note in the Study 2 Discussion that this could be an avenue for future research: “The significant effect of transgression type may be limited to the specific transgressions that we chose. To test the robustness of this effect, future research can use a broader range of transgressions, and ensure that harm and purity transgressions are matched on severity.” (p. 18: lines 414–417).*

**The authors note that the current research focuses on everyday transgressions rather than the kinds of severe ones that are typically studied. It would be helpful to explicitly lay out why these contexts might lead to different findings, and to describe how the current results map onto what has been observed for more serious transgressions. I also wonder how adults would reason about more serious transgressions using the current paradigm.**

*We agree with the Reviewer that different types of transgressions might lead to different findings. In the General Discussion, we have now noted that there is evidence that judgments of transgressions are heavily influenced by the depravity or severity of the action, as well as the effect on the victim. Specifically, we state, “Unlike most previous studies that have investigated severe transgressions such as murder and harsh sentences such as capital punishment [3, 19, 21], the current research focused on more everyday moral transgressions (e.g., throwing rocks on a playground) and more typical sanctions (e.g., time-out). Because judgments of transgressions are heavily influenced by the depravity or severity of the action, as well as their effects on victims [e.g., 58], these judgments may also be differentially affected by a perpetrator’s background characteristics. Thus, future research could use the current ‘switched-at-birth’ paradigm to examine how adult participants may make judgments of punishment about transgressions of a range of severity, in order to see if the null effects observed in this research hold across such transgressions with a broad range of perceived depravity and negative consequences” (p. 27: lines 615–624).*

**It is interesting that people can use information about genes to help them assess a range of characteristics (like dangerousness, attributions of desire to commit immoral acts, and perceptions of controllability) but not punishment. I wonder whether the lack of effects hold when looking only at the more general version of the punishment question (“How much trouble should Jane/Fred get into for hurting the other children?”).**

*The lack of effects do hold for this specific question. Results are as follows:*

*Study 1 – Genes: F(2,158) = .261, p = .771, η^2^_p_ = .003; Environments: F(2,158) = 1.222, p = .297, η^2^_p_ = .015; Genes X Environments: F(4,158) = .510, p = .728, η^2^_p_ = .013.*

*Study 2 – Genes: F(1,132) = 1.348, p = .248, η^2^_p_ = .010; Environments: F(1,132) = 2.366, p = .126, η^2^_p_ = .018; Genes X Environments: F(1,132) = 2.125, p = .147, η^2^_p_ = .016.*

*Study 3 – Genes: F(1,140) = .318, p = .574, η^2^_p_ = .002; Environments: F(1,140) = .253, p = .616, η^2^_p_ = .002; Genes X Environments: F(1,140) = 2.710, p = .102, η^2^_p_ = .019.*

**Outcomes are important for punishment recommendations, as is suggested, but there are other possibilities too. It could be that genetic factors have effects that go in opposite directions, as might be the case if attributions of desire to commit immoral acts alone would lead to increases in punishment recommendations, and decreases in perceptions of controllability alone would lead to decreases in punishment recommendations. More explanation is also needed regarding how the lack of punishment effects can be understood in light of the evidence mentioned in the introduction that characteristics that are seen as linked to genetic influences can mitigate punishment assessments.**

*We now acknowledge some of these other possibilities. Specifically, we now state, “Future research will need to further confirm these null effects – for instance, by testing whether they are due to nullifying competing effects across participants (with some participants treating genetic factors as mitigating and some participants treating genetic factors as aggravating) [24]. Although we did not find compelling evidence of genetic information acting as a ‘double edged sword’, it will be important for future work to help reconcile our findings with some of the positive effects that have been previously uncovered by other researchers [e.g., 26–29]” (pp. 26–27: lines 608–614).*

**Reviewer #2:**

**The authors neglected a fairly large corpus of research conducted by Appelbaum and colleagues that has examined this exact issue. In particular, his 2016 piece in the Journal of Law and Biosciences examined the impact of biological influences on misbehavior in non-criminal settings and in children. Some discussion of that study (and the others conducted by his group) is warranted. There are also numerous studies published in basic social psychology journals that examine how genetics affect attributions made by laypeople.**

*We have now included mention and discussion of both the Appelbaum JLB article and one of their other pieces, and their relevance to our study, in the Introduction and General Discussion (p. 3: lines 90–92; p. 4, lines 104–108; and p. 26: lines 594–599).*

**Study 1 had 9 cells and 201 participants. That amounts to 22 participants per cell. On its face, that is drastically underpowered. Especially in light of the fact that the previous literature finds null or small effects, the sample size must be large to be able to detect an effect assuming it does exist. (It also makes observed “statistically significant” effects unlikely to be true effects; see Button et al Nature Neuroscience for an explanation of this principle.) This sample size is inadequate to study this phenomenon. The problem is even more dramatic in the subsequent studies. The studies should be re-ran with 5 to 6 times the amount of participants, at least. If the findings replicate, then I would recommend publication.**

*We have now included power analyses in the Participants sections of all three studies (p. 6: lines 157–160, pg. 14: lines 328–331, p. 20: lines 462–465). Those analyses indicate that, for each study, we have more than enough power to detect medium-sized effects (fs >= .25, power >= .80, alpha = .05).*

*We considered re-running the studies with much larger samples, in order to detect very small effects (e.g., fs = .1). However, we realized that would amount to hunting for tiny effects, and that would be a deviation from the goals that we had at the outset of this project: to examine whether perpetrators’ backgrounds have meaningful influences on participants’ inferences about perpetrators and punishment administered to perpetrators. We are not particularly interested in detecting very small effects because it is not clear that those effects would have practical significance (e.g., in court), and it is unclear that very small effects (e.g., accounting for 1% of the variance in punishment decisions) would help to settle any theoretical debates.*

*It is also worth noting that our experimental manipulations (of genetic background and environmental background) were strong enough and our power was strong enough that we were able to detect effects for several DVs (e.g., character), even though we consistently did not find effects on punishment decisions.*

*We acknowledge the replication issues in psychological research. We hope that the new power analyses and the fact that several of our findings indeed replicate across studies help to alleviate this concern with the current work.*

**Reviewer #3:**

**I found it striking that the results of studies 1 and 3 were so similar, while the pattern of results in study 2 was quite different. However, rather than highlighting the different pattern of results for study 2, the authors seem to saw in the first sentence of study 2's discussion section that the results were similar, which I found surprising. It might be more effective for the authors to explore/explain why study 2 might have had such a different pattern of results. It's interesting that in all of the studies, judgments of intentionality and free will were significantly affected by either genetic or environmental explanations, but seemingly never by both, whereas character ratings were seemingly affected by both kinds of explanations. The authors may wish to explore/discuss why this may be.**

*We agree that it is important to highlight the slightly different pattern of results for Study 2, and we thank the reviewer for calling our attention to this issue in our previous framing of our discussion. We have added a more complete acknowledgment of this discrepancy (in Studies 1 and 3, bad genes elevated judgments of intent, while in Study 2, growing up a positive rearing environment elevated judgments of intent) in both the discussion of Study 2 (p. 18: lines 405–417) and in the General Discussion (pp. 24–25: lines 560–571) before highlighting any similarities. However, we believe we cannot conclude anything definitive from these mixed findings between studies, and so we avoided making any speculative claims here (see p. 18: lines 414-417, and pp. 24–25: lines 564–568).*

**Also interesting is the finding that punishment judgments, while sensitive to the amount of harm caused by the wrongdoing, do not seem to be responsive to genetic or environmental explanations. The lack of an effect on punishment is similar to what others have found (see the work of Paul Appelbaum and the work of Steve Heine).**

*We now discuss the similarity of our results to those found by Paul Appelbaum and work by Steven Heine (p. 26: lines 596–599), both of which found null effects for punishment decisions. Additionally, Heine also found that genetic information influenced judgments about the perpetrator’s control and likelihood of reoffense, but these judgments did not translate to punishment decisions.*

**Reviewer #4:**

**There were two elements of the paper that I found lacking. I elaborate below:**

**1) The design (as authors acknowledge) is targeted to a developmental population. Authors argue that the design works equally well for adults vs. children, but I do not agree. For one thing, the perpetrator is a child, which for an adult study is an odd choice. It is not clearly wrong, however; I think one could make the argument that choices about punishment for a child vs. adult are still interesting and relevant. But there is another larger problem here: The genetic background manipulation relies entirely on inference. Participants are not told at all about bad or good genes; instead they are told about “something in the blood and brain” that makes the biological parents mean or nice. Participants then must infer that the meanness or niceness is genetic, and that the “bad” or “good” genes are heritable. Though this phrasing makes sense if there were child subjects as part of the project, for a fully adult study, it’s just not a sensible phrasing. Authors argue that this simplified language is appropriate, but again I do not agree, or at least I’m not sure without a manipulation check. Specifically, do adults interpret these scenarios as involving genes? Or, if the authors wanted to develop a manipulation check that could be used with children, do participants interpret these scenarios as involving a feature that passes down from parent to child?**

*We agree that saying that something is “in the blood and brain” is not exactly the same as saying that something is “genetic”; at least, it is not the same from a scientific standpoint. However, we chose this specific wording for several reasons. For one, it helps to convey the key idea that the circumstances are biological in nature (and thus, the manipulation of those circumstances is clearly distinct from our manipulation of evidence about the perpetrators’ external, social contexts). Whereas the general population of adults may or may not understand genetics, most will understand what it means for something to exist in the blood and brain (and if future studies are conducted with child participants, this exact methodology and language can still be used, allowing for direct comparisons to these adult data, something that we now elaborate on in the General Discussion).*

*Given the Reviewer’s important point, we wanted to verify our assumption that adults interpreted our language as indicating genetic inheritance by looking more closely at the current data. Specifically, to gauge the extent to which participants interpreted the information in the vignettes as indicating inherited, genetically-influenced traits, two coders (who were blind to hypotheses) coded all open-ended responses according to whether each participant utilized each of the following categories of terms:*

*(1) “Genes”, “Genetic”, “DNA”, or “Inherited”*

*(2) “Blood”, “Brain”, “Heart”, “Neurological” (or other close synonyms of any of these terms)*

*(3) Other More Ambiguous Biological Terms: “Biology”, “Biological”, “Instinct”, “Disease”, “Born with”, “Medical Condition”, “Nature”, “Related to [Birth Parents]”, “Inherently”, “In His/Her Body” (or other close synonyms of any of these terms)*

*(4) “Nurture”, “Environmental”, “Upbringing”, “Reared”, “Raised”, “Taught”, “Exposed”, “Grew Up In/With”, “Sees This At Home”, “Treatment at Home”, “Conditioned”, “Has Been Shown” (or other close synonyms of any of these terms)*

*This yielded the following results (collapsed across Conditions):*

|  | ***Genes/DNA*** | ***Blood/Brain*** | ***Other Biology*** | ***Nurture*** |
| --- | --- | --- | --- | --- |
| ***Study 1*** | *14.97%* | *7.19%* | *7.19%* | *25.15%* |
| ***Study 2*** | *13.57%* | *9.29%* | *4.29%* | *29.29%* |
| ***Study 3*** | *9.46%* | *4.05%* | *4.05%* | *22.97%* |

*Additionally, when participants provided open-ended explanations in the “bad genes” conditions, references to genes/DNA increased for all studies: Study 1 goes up to 33.90%, Study 2 goes up to 25.37%, and Study 3 goes up to 13.43%. To us, this strongly suggests that participants frequently interpreted information about “something in the blood and brain” as indicating an inherited genetic condition. Furthermore, the manipulation of the quality of the biological background influenced inferences that behavior was a function of genetics/DNA.*

*Even if some participants did not infer that “something in the blood and brain” is necessarily genetic, this language reduces participants’ need to make other inferences. Genes influence individual behavior by virtue of the effects that they have on specific biological systems (e.g., the blood and brain). It is unclear that lay adults will understand exactly how genes function to ultimately influence traits or behavior. Thus, telling participants about those biological systems bridges the key chain of inferences: genes 🡪 biological systems 🡪 phenotypes and behavior. Because we specify the biological systems at play (blood and brain, thus highlighting the more proximal causes of perpetrators’ behavior), this could potentially strengthen the effects of evidence about biological circumstances on our dependent variables; yet we still found no effects of genetic/biological information on punishment decisions.*

**2) Findings are not consistent across the three studies; effects for genes and environment are not reliably present. (Genes had effects for Studies 1 and 3 and environment mostly did not; environment had effects for Study 2 and genes mostly did not.) I don’t want to dwell too long on this, as this is not the fault of the authors and was not a flaw of the studies (though I suppose it might be partly a result of a weak or variably interpreted manipulation, see above). Ultimately, we just can’t conclude anything unambiguously from the mixed findings that emerged. As well, authors face the challenge of presenting a null finding as one of the most important results of the studies, which is just inherently a hard thing to do. This probably weakens the overall appeal of the paper. Again, though, I see this as a minimal weakness.**

*We agree with the Reviewer that more attention should be paid to the differences of effects between Studies 1 and 3 with those of Study 2 (also see our response to Reviewer #3). Thus, we added a more complete acknowledgment of this discrepancy (in Studies 1 and 3, bad genes elevated judgments of intent, while in Study 2, growing up a positive rearing environment elevated judgments of intent) ) in both the discussion of Study 2 (p. 18: lines 405–417) and in the General Discussion (pp. 24–25: lines 560–571). We agree that we cannot conclude anything from these mixed findings between Studies, and avoided any speculative claims here (see p. 18: lines 414-417, and pp. 24–25: lines 564–568). However, we disagree that findings across all three studies are overall inconsistent. While we cannot conclude anything with certainty about genetic and environmental influences on mental states, we found that character assessments were particularly malleable to information about nature and nurture. Furthermore, none of these judgments translated into punishment decisions across all three studies. Although null, we rigorously tested the potential of genetic and environmental influences on punishment decisions across a variety of moral transgressions, and thus found the lack of influence to be striking, and important to present and discuss.*

**-If space permits, I would like to see the choice options (or the anchors) for the Likert scales included somewhere in the paper, rather than in supplementary materials.**

*The choice options for the Likert scales are now included for each question in the Study 1 Design and Procedure section (p. 7: lines 184–185; p. 8: lines 188–190 and 190–192).*

**-Was gender included as a factor in initial analyses? If gender was not significant, stating this would be helpful.**

*We had no specific predictions about Gender, and examining gender differences was not an a priori goal of these studies, so it was not included in our analyses. We have rerun the analyses with Gender included as a factor, and it is consistently non-significant, with a single exception (Study 2, Character: p = .048). We do not report these analyses in the manuscript because they do not speak to our research questions, and because they are post-hoc (rather than planned) analyses.*

**-As noted in my summary, I think there is a mistake in Figure 4, for the Punishment variable. The text describes punishment as being harsher for harm vs. purity violations in Study 2, which is the opposite pattern from that depicted in Figure 4.**

*We are extremely grateful to the reviewer for catching this mistake (the previous Punishment graph erroneously displayed the means from the Character DV), which we have remedied. We have triple-checked the results in the rest of the manuscript to ensure that there were no additional errors.*

**-Could authors clarify in the text what exactly was being tested in the essentialism measures they used? (What kind of essentialism was being measured? Global? Essentialism of aggression/criminality/prosociality? Some other kind of social essentialism?)**

*We have clarified which set of essentialism measures were used in each study, as well as what each set of measures tested with regard to the area or type of essentialism (see Footnote 2 on p. 9). Study 1 used validated items measuring overall genetic and social essentialism (Keller, 2005; Rangel & Keller, 2011) and the two subsequent studies using a measure of overall genetic essentialism (Keller, 2005) in Study 2 and adding an additional measure gauging discreteness, informativeness, and biological basis (Bastian & Haslam, 2008) in Study 3. We have also now included the essentialism items used in each study in the Supplementary Materials.*
